# Systemic regulation of rheumatoid arthritis by mesenchymal stem cells: from immune homeostasis to microbiota modulation

**DOI:** 10.3389/fimmu.2026.1835684

**Published:** 2026-07-14

**Authors:** Liujiayu Li, Jieling Wu, Yutong Wu, Juan Liu, Lu Feng, Shangfu Xu, Yuying Wang, Limei Yu

**Affiliations:** 1 Key Laboratory of Cell Engineering, Affiliated Hospital of Zunyi Medical University, Zunyi, China; 2 Key Laboratory of Cell Engineering of Guizhou Province, Affiliated Hospital of Zunyi Medical University, Zunyi, China; 3 Collaborative Innovation Center of Tissue Damage Repair and Regenerative Medicine, Zunyi Medical University, Zunyi, China; 4 Key Laboratory of Basic Pharmacology of Ministry of Education, School of Pharmacy, Zunyi Medical University, Zunyi, China

**Keywords:** clinical translation, gut microbiota, gut-MSC-immune axis, immunomodulation, intestinal-articular axis, mesenchymal stem cells, rheumatoid arthritis, treg/Th17 balance

## Abstract

Rheumatoid arthritis (RA) is an autoimmune disease characterized by chronic synovitis and progressive joint destruction. Although conventional therapies effectively alleviate clinical symptoms, they are largely incapable of reversing irreversible joint damage and the underlying immune dysregulation. Mesenchymal stem cells (MSCs), with their dual capabilities in immunomodulation and tissue repair, have emerged as a promising new therapeutic strategy. This review delineates how MSCs and their derived extracellular vesicles exert multifaceted anti-inflammatory and immunosuppressive effects in RA. Locally, these mechanisms include modulating the Treg/Th17 cell balance, inhibiting the proliferation and invasion of fibroblast-like synoviocytes, and regulating the RANKL/OPG system to suppress osteoclast activity, while orchestrating the repair and regeneration of damaged joints primarily through the secretion of trophic factors and paracrine regulation of local bone metabolism. Importantly, beyond these local effects, this review highlights the novel ‘gut-MSC-immune’ axis, elucidating the potential pathway through which MSCs might systemically modulate immune homeostasis by influencing gut microbiota composition and mucosal barrier integrity. Although preliminary clinical trials have demonstrated a favorable safety profile and potential therapeutic feasibility of MSC-based interventions, its therapeutic outcomes are significantly influenced by patient heterogeneity and the hostile inflammatory microenvironment. To address these clinical bottlenecks and overcome interpatient therapeutic heterogeneity, we discuss the synergistic potential of microbiota-targeted interventions combined with MSC therapy. Finally, key priorities for future research are proposed, including standardizing MSC preparation protocols, optimizing administration regimens, and validating long-term efficacy and safety via large-scale multicenter clinical trials—efforts that will ultimately accelerate the clinical translation and clinical application of precision MSC-based therapy for RA.

## 1 Introduction

Rheumatoid arthritis (RA) is a chronic, progressive autoimmune disease primarily affecting synovial joints, with potential extra-articular manifestations involving the skin, eyes, heart, kidneys, and lungs (1). Ultimately, patients may develop joint failure characterized by cartilage damage and severe impairment of tendons and ligaments (2). RA causes progressive disability and premature death, with systemic complications being the primary driver of increased morbidity and mortality in affected patients.The global prevalence of RA is estimated at 0.5%-1.0% (3). It can affect people of all ages and genders, but it is more common in older people, and women are also more susceptible to the condition (4). As a polyarticular arthritis, RA most commonly affects the joints of the hands, feet, and knees, and may involve the spine, but typically spares the distal interphalangeal joints (5). Extra-articular involvement is a critical clinical feature, potentially leading to cardiac, pulmonary, renal, and intestinal pathologies, as well as widespread vasculitis, complications that can be directly fatal (6).

Current disease-modifying antirheumatic drugs (DMARDs) primarily target inflammation and pain management; however, they impose a substantial economic burden and lack the regenerative capacity to repair established joint damage (7). In accordance with the latest EULAR/ACR guidelines, DMARDs are systematically categorized into conventional synthetic DMARDs (csDMARDs, e.g., methotrexate, leflunomide), biologic DMARDs (bDMARDs, e.g., TNF-α inhibitors), and targeted synthetic DMARDs (tsDMARDs) such as Janus kinase (JAK) inhibitors (8) First-line agents typically include hydroxychloroquine, methotrexate, leflunomide, and sulfasalazine (9). As traditional symptomatic agents, non-steroidal anti-inflammatory drugs (NSAIDs) exhibit limited efficacy due to their systemic administration and lack of specificity, and are associated with significant risks of gastrointestinal complications and nephrotoxicity—side effects well-supported by clinical evidence (10, 11).

Mesenchymal stem cells (MSCs) are a type of adult stem cell capable of self-renewal and multipotent differentiation, and are widely found in various tissues of animals and humans (12). The key biological properties of MSCs include low immunogenicity, anti-inflammatory effects, anti-apoptotic properties, and immunomodulatory capabilities (13). They are widely used in therapeutic applications and research on various diseases, including RA (14). Therefore, thanks to its powerful immunomodulatory and regenerative properties, it has become a highly promising cell therapy for the treatment of RA, While preclinical investigations have provided foundational insights into the mechanisms by which MSCs modulate immune responses, their clinical translation for RA is still in its nascent stages (15). Although early-phase clinical evaluations have preliminarily explored the safety and potential feasibility of MSC-based interventions, the current body of clinical evidence remains heterogeneous and far from definitive (16). Further large-scale, standardized trials are essential to clarify their therapeutic efficacy and long-term safety profiles in RA patients (17).

Given the unmet clinical needs of conventional therapies, MSCs have emerged as a prominent research focus in RA treatment. This review aims to provide a focused overview of the disease characteristics and pathogenesis of RA, and to synthesize the current research progress regarding the mechanisms of MSC action. Specifically, we explore the local immunomodulatory mechanisms of MSCs within the joint microenvironment and highlight their systemic regulation through the novel ‘gut-MSC-immune’ axis. Furthermore, by discussing current clinical translational challenges, we underscore the potential of microbiota-MSC combination therapies for precision RA management. Building upon existing MSC-in-RA literature, this review incorporates a systemic immune-metabolic perspective to provide a broader understanding of MSC therapies, aiming to offer new clues for addressing current clinical bottlenecks.

## 2 Pathogenesis of RA

### 2.1 Immune dysregulation and the initiation of inflammation

The disruption of immune homeostasis is the core driving force behind the onset and progression of RA, characterised by a widespread loss of self-tolerance and a profound imbalance in immune cell subsets. At the mechanistic level, impaired regulatory T cell (Treg) suppressive function constitutes a key cornerstone of this homeostatic imbalance; the decline in their numbers and function is one of the critical factors in the breakdown of self-tolerance (18). Functional defects in Treg cells allow pro-inflammatory cell subsets, particularly M1 macrophages and Th1 cells, to be extensively recruited and to trigger inflammatory signalling cascades, whilst highly activated Th17 cells and their characteristic effector cytokine, IL-17, play a dominant role in the initial induction phase of the disease (19). It is worth noting that this immune dysfunction is by no means confined to the local joint site, but is deeply involved in the systemic inflammatory network (20), and is closely associated with pathological processes such as intracellular metabolic reprogramming and abnormal biomechanical transmission (21). Furthermore, the gut microbiota (including the gut virome) has also been shown to be extensively involved in the early pathogenesis of RA through distant immune regulatory mechanisms (22).

### 2.2 The amplification cascade of the cytokine network

The aforementioned upstream immune imbalance further directs and triggers a massive cytokine cascade, thereby causing chronic and persistent pathological damage to joint tissues (23). A wealth of robust preclinical and clinical studies has clearly demonstrated that pro-inflammatory cytokines—particularly tumour necrosis factor-α (TNF-α), interleukin-1 (IL-1) and interleukin-6 (IL-6)— —play a dominant role in the amplification and spread of the inflammatory response in RA, with IL-6 being particularly critical in inducing and maintaining chronic local and systemic inflammation. It acts on a wide range of target cells (including synovial fibroblasts) by activating the JAK-STAT pathway via classical signalling routes, as well as through trans-signalling, thereby inducing and sustaining chronic local and systemic inflammation (24), Furthermore, IL-6 is capable of JAK-dependently activating the transcription co-activator Yap, thereby inducing Snail expression and promoting the invasive phenotypic transformation of synovial fibroblasts; this constitutes an important cellular basis for synovial hyperplasia and bone erosion in RA (25). However, these inflammatory mediators do not act in isolation; they not only recruit additional peripheral immune cells to infiltrate the synovial tissue but also interact and communicate frequently with neutrophil extracellular traps (NETs) and reactive oxygen species (ROS), thereby forming a self-reinforcing inflammatory vicious cycle (26). Nevertheless, clinically, approximately 50% of patients show no response to biologic therapies targeting these classic cytokines (27), suggesting the existence of a more complex cytokine network underlying this phenomenon. Among these, the Janus kinase (JAK)-mediated intracellular signalling pathway, as a key regulatory node downstream of this network, has also become an important target for current drug interventions (28).

### 2.3 FLS activation and synovial remodeling

Driven by a persistent cytokine storm, synovial fibroblasts (FLS) undergo a profound phenotypic transformation, becoming the key effector cells in joint damage. Activated RA-FLS exhibit tumour-like invasive characteristics, including uncontrolled proliferative capacity, anti-apoptotic properties, highly potent migratory and invasive activity, and significant metabolic reprogramming (29). Under physiological conditions, healthy FLS inherently possess a ‘regulatory’ phenotype that inhibits T-cell activation, promotes resolution of inflammation, and maintains immune tolerance (30); however, in the pathological environment of RA, this protective mechanism is entirely absent. Instead, FLS that have shifted to a ‘pro-inflammatory’ phenotype begin to autonomously secrete large amounts of TNF-α and IL-6, establishing a self-sustaining inflammatory circuit that directly leads to abnormal remodelling of synovial tissue and the formation of vascular pseudomembranes (30). Interestingly, FLS from different anatomical sites exhibit unique, site-specific biological characteristics, which explains at the molecular level why RA exhibits a clinical tendency to affect the small joints of the hands (31). Furthermore, these pathogenic FLS display high expression of various activation markers at the molecular level, such as the significant expansion of the HLA-DR^+^CD90^+^ subpopulation (driven by IFN-γ) (32). This pathogenic state is characterised at the molecular level by high expression of fibroblast activation protein (FAP) and is further exacerbated by severe downregulation of peroxisome proliferator-activated receptor-γ (PPAR-γ), as the latter plays a key role in suppressing abnormal proliferation and excessive activation of FLS under normal physiological conditions (33). Furthermore, through single-cell transcriptomics and spatial transcriptomics analyses, researchers have identified several functionally distinct FLS subpopulations, such as the CD142^+^ fibroblast subpopulation located in the lining layer, whose distribution and abundance in the synovium of RA are altered, and which is directly involved in synovial hyperplasia and matrix remodelling (34).

### 2.4 Skewing of the RANKL/OPG axis

The prolonged state of high inflammation in the synovial tissue and adjacent immune compartments directly disrupts the delicate balance of bone metabolism. This balance is physiologically regulated by three key factors: the receptor activator of nuclear factor κB (RANK), its ligand (RANKL), and the decoy receptor osteoprotegerin (OPG). In the microenvironment of RA joints, the RANKL/OPG ratio undergoes a significant pathological elevation, thereby acting as a decisive switch triggering abnormal osteoclastogenesis and periarticular bone loss. Studies have shown that the accumulation of NETs in synovial fluid is strongly positively correlated with an elevated RANKL/OPG ratio, with patients exhibiting severe bone erosion displaying higher concentrations of NETs (35); this pathological chain has been further validated in *in vivo* experiments: inhibiting NET formation through DNase degradation or Padi4 gene knockout significantly alleviates bone loss in arthritic mice (36). Furthermore, evidence suggests that an imbalance in CD4+ T cell subsets is crucial in RA-induced bone destruction. Th17 cells induce the expression of RANKL in synovial fibroblasts and osteoblasts by secreting cytokines such as IL-17, whilst Treg cells suppress osteoclastogenesis by secreting IL-10 and TGF-β (37). In the RA microenvironment, an elevated Th17/Treg ratio further increases the RANKL/OPG ratio, thereby synergising with NETs to drive excessive osteoclast activation (37). Concurrently, other auxiliary mechanisms play significant roles; among these, immune complexes can directly or indirectly enhance the sensitivity of osteoclast precursors to RANKL by activating Fcγ receptors (FcγR) (38), whilst high expression of TLR2, in conjunction with specific α2, 3 sialylation modifications, converges upon and acts on the RANKL-RANK signalling axis, significantly accelerating osteoclast fusion and the local bone resorption process (39, 40).

### 2.5 Osteoclast-driven irreversible joint destruction

**Figure 1:**
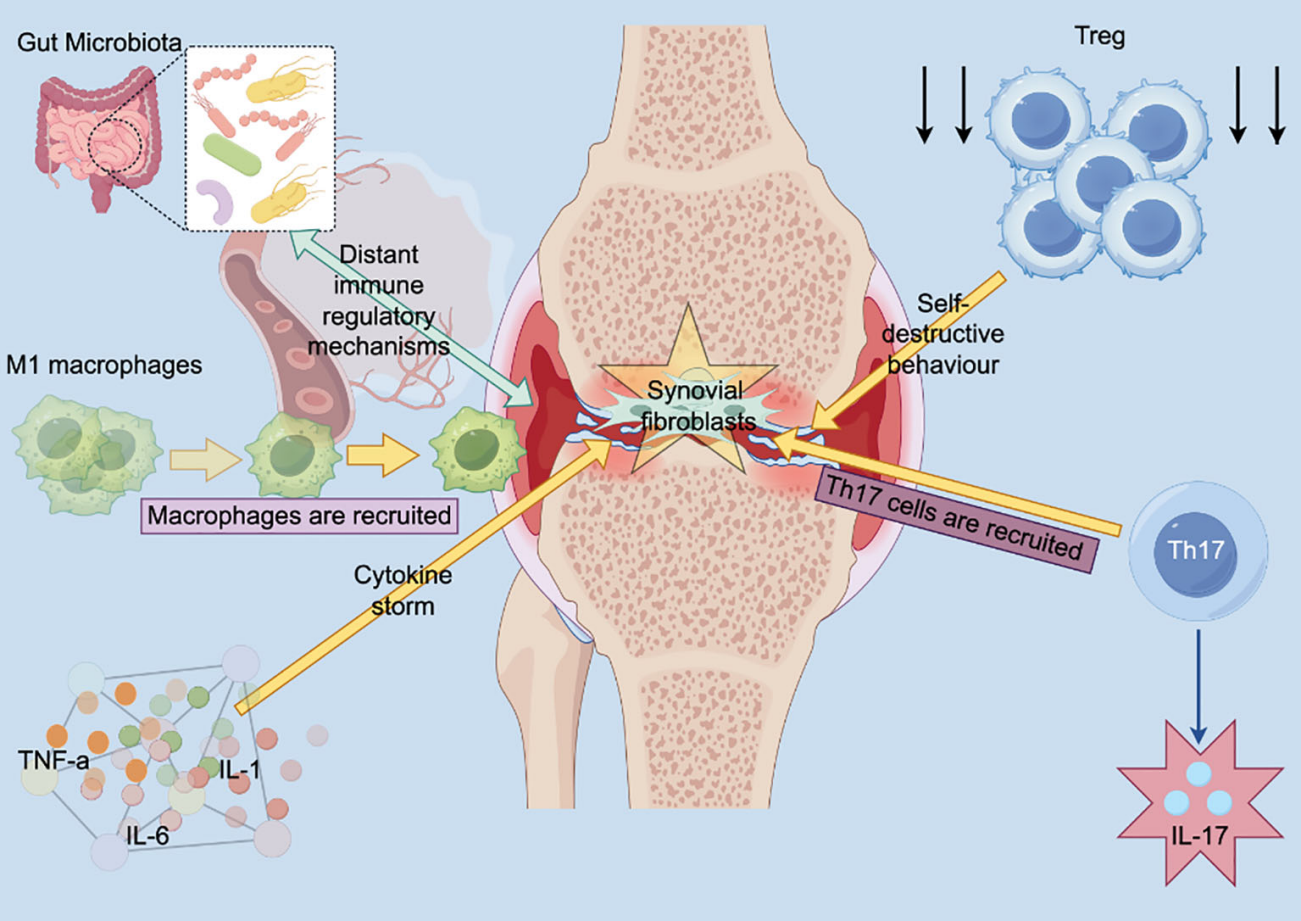
The core pathogenic mechanisms and inflammatory cascade in RA. The disruption of immune homeostasis and subsequent systemic inflammation drive the progression of RA. (1) Immune Dysregulation: A profound decline in the number and suppressive function of regulatory T cells (Treg) leads to the breakdown of self-tolerance, initiating self-destructive behaviours. (2) Inflammatory Cell Recruitment: Pro-inflammatory subsets, notably M1 macrophages and Th17 cells, are extensively recruited to the joint. Th17 cells and their effector cytokine, IL-17, play a dominant role in the initial disease phase. (3) Distant Immune Regulation: The gut microbiota is deeply involved in early RA pathogenesis through distant immune regulatory mechanisms, highlighting the systemic nature of the disease. (4) Cytokine Storm and Amplification: The upstream immune imbalance triggers a massive cytokine cascade (predominantly TNF-α, IL-1, and IL-6). These pro-inflammatory cytokines act on local target cells, such as synovial fibroblasts, sustaining chronic inflammation and driving progressive joint tissue damage.

As the only specific multinucleated cells in the body capable of bone resorption, osteoclasts play a central role in the destruction of joints in RA. Persistent autoimmune reactions and chronic inflammation within the joints of RA patients create a highly ‘hostile’ microenvironment, leading to the excessive activation and accelerated proliferation of osteoclasts, accompanied by impaired osteoblast function. The imbalance between these two cell types ultimately results in a complete breakdown of bone remodelling homeostasis, manifesting as marginal bone erosion, periarticular bone loss and systemic osteoporosis (37).This pathological osteoclastogenesis is not driven by a single factor, but rather by a complex network jointly regulated by local inflammatory signals (such as IL-1β, TNF-α, M-CSF, etc.), genetic susceptibility, and epigenetic modifications (41). Furthermore, immune complexes (IgG) can promote osteoclast differentiation via Fcγ receptors (FcγRs), and certain FcγR signalling molecules (such as FcγRI) have been identified as potential therapeutic targets (41). It is worth noting that neutrophil extracellular traps (NETs), acting as inflammatory mediators in RA, can also stimulate bone loss by enhancing osteoclastogenesis, suggesting that blocking NETs may represent a novel strategy for combating bone erosion (35). Current and future translational medical research must focus on dual therapeutic strategies that simultaneously target osteogenesis and its upstream inflammatory cascades. This will not only help to halt the progressive destruction of joint structures, thereby improving clinical outcomes such as deformity, functional impairment and reduced quality of life, but also holds promise for overcoming the current limitation of disease-modifying antirheumatic drugs (DMARDs), which can control inflammation but struggle to completely halt bone erosion (**Figure 1**).

## 3 The mechanism of MSC-based treatment for RA

The core pathological network of RA is driven by three key mechanisms: systemic immune homeostasis imbalance, abnormal proliferation of fibroblast-like synoviocytes (FLS), and progressive destruction of bone and cartilage. MSCs and their derivatives demonstrate significant therapeutic potential by intervening in these pathological processes through multi-targeted mechanisms. Here, we elucidate the specific mechanisms of action of whole MSCs, source-specific MSC lineages, and MSC-derived extracellular vesicles (MSC-EVs), across three biological dimensions: immune regulation, synovial suppression, and bone and cartilage repair.

### 3.1 Anti-inflammatory and immunomodulatory effects

#### 3.1.1 The direct immunomodulatory effects of MSCs

MSCs can directly influence the differentiation fate of CD4^+^ T cells through cell-cell contact and the secretion of soluble factors (such as PD-L1). For example, MSC-derived exosomes overexpressing PD-L1 (Exo-PD-L1) can directly induce apoptosis in Th17 cells whilst upregulating the proportion of Treg cells, thereby restoring the Treg/Th17 balance (42) Furthermore, endoplasmic reticulum stress-pretreated MSCs (ERS-MSCs) can significantly suppress follicular helper T cells (Tfh) in RA via the cyclooxygenase pathway, thereby exerting an immunoregulatory effect (43). Additionally, studies have found that in a CIA model, MSCs can promote Treg differentiation and suppress Th17 generation through multiple pathways. For example, studies have shown that a mesenchymal stem cell-derived nanodrug delivery system (NDDS) can significantly promote Treg differentiation in the spleens of CIA mice, whilst reducing the proportion of Th17 cells, thereby reversing the Treg/Th17 imbalance and alleviating synovial hyperplasia and cartilage damage (44). Furthermore, MSCs not only regulate T-cell subsets but also influence other immune cells to exert immunomodulatory effects. MSCs can promote the polarisation of macrophages towards an anti-inflammatory phenotype and induce T-cells to differentiate in a regulatory direction, thereby downregulating pro-inflammatory factors and upregulating anti-inflammatory factors (45). These effects collectively constitute the core mechanism of direct immunoregulation by MSCs, namely correcting the differentiation imbalance of CD4^+^ T-cell subsets at the root and restoring immune homeostasis. MSCs can also regulate dendritic cells (DCs) to restore their immunosuppressive function; tolerant dendritic cells suppress autoimmune responses by differentiating into Tregs, and MSCs are capable of promoting this process of immune tolerance restoration (46).

#### 3.1.2 Immunomodulatory specificity of MSCs from different tissue sources

The immunomodulatory targets and efficacy of MSCs exhibit significant heterogeneity depending on their tissue of origin. Bone marrow-derived mesenchymal stem cells (BM-MSCs) were among the first cell types used in RA research; they directly target synovial T cells by secreting PD-L1-enriched exosomes (Exo-PD-L1), thereby enhancing immunosuppressive functions. BM-MSCs can also modulate the function of dendritic cells (DCs), inducing their differentiation into tolerant dendritic cells (tolDCs), thereby promoting Treg differentiation and restoring immune tolerance (42).

Human umbilical cord mesenchymal stem cells (UC-MSCs) focus on the systematic regulation of key transcription factors. They are capable of downregulating the expression of RORγt, a key transcription factor in Th17 cell differentiation, whilst upregulating Foxp3, a core factor in Treg cell differentiation. This precisely reduces the proportion of Th17 cells in the spleens of CIA mice and increases Treg levels, thereby directly improving systemic immune tolerance (47), Furthermore, we observed that UC-MSCs can induce apoptosis in leukaemia cells by secreting tumour necrosis factor-α-related apoptosis-inducing ligand (TRAIL), and block the cell cycle by secreting indole-3-ol-2, 3-dioxygenase (IDO). Although these mechanisms have been reported in tumour models, they suggest the potent immunosuppressive potential of UC-MSCs (42); Human adipose-derived mesenchymal stem cells (AT-MSCs), on the other hand, primarily act on the functional activation of pathogenic CD4^+^ T cells. Studies have shown that AT-MSCs can reduce the frequency of granulocyte-macrophage colony-stimulating factor (GM-CSF)-positive CD4^+^ T cells in peripheral blood and the spleen, whilst simultaneously promoting the targeted infiltration of Tregs into sites of synovial inflammation, thereby achieving bidirectional immunosuppression from the systemic to the local level (48). In addition to UC-MSCs and AT-MSCs, MSCs from other sources have also demonstrated unique characteristics in the treatment of RA. Studies have clearly indicated that mesenchymal stem cells (SF-MSCs) isolated from the synovial fluid of patients with RA possess significant disease-specific features. Prolonged exposure to a chronic inflammatory microenvironment (including hypoxia and pro-inflammatory cytokines) leads to impaired proliferative capacity, accelerated cellular senescence, diminished immunomodulatory properties, and reduced anti-arthritic potential in SF-MSCs within RA animal models (49), Specifically, compared with patients with early-stage RA, SF-MSCs isolated from the synovial fluid of patients with longer disease duration more frequently exhibit cellular senescence, diminished immunomodulatory function, and loss of anti-arthritic potential. Further studies have revealed that even in patients with early-stage RA, when SF-MSCs are placed in a chronic inflammatory environment simulating RA joints (such as hypoxia and pro-inflammatory factor stimulation), they undergo cellular senescence and a decline in immunomodulatory function similar to that observed in late-stage RA (50). These findings suggest that RA patients’ own SF-MSCs are functionally impaired due to prolonged exposure to the disease microenvironment. If used for autologous MSC therapy, their immunomodulatory activity and tissue repair capacity may be significantly lower than that of MSCs from healthy donors; therefore, the efficacy and safety of autologous MSC therapy require careful evaluation.

#### 3.1.3 The independent immunomodulatory effects of MSC-EVs

The independent immunomodulatory effects of MSC-EVs To circumvent the senescence and functional decline of live autologous MSCs in the hostile RA microenvironment, MSC-EVs have emerged as a promising cell-free alternative. As key carriers of the paracrine pathway of MSCs, MSC-EVs possess the advantages of low immunogenicity and the ability to readily traverse biological barriers, enabling them to exert immunomodulatory functions independently of intact cells. Research has shown that human dental pulp MSC-derived exosomes (GMSC-EVs) can specifically home to inflamed joints in RA following intravenous administration. By utilising the encapsulated miR-148a-3p, they precisely target and regulate the IKKβ/NF-κB signalling pathway, blocking inflammatory signals at the transcriptional level and thereby effectively restoring the Treg/Th17 balance within the joint cavity (51) Furthermore, in addition to directly regulating the Treg/Th17 balance, MSC-EVs can inhibit inflammatory cascades at multiple upstream nodes; MSC-EVs can modulate the functions of innate and adaptive immune cells, including inhibiting the abnormal maturation and activation of dendritic cells and macrophages. Through this mechanism, they are able to ‘reduce the release of pro-inflammatory factors such as IL-6 and TNF-α’, thereby blocking the positive feedback loop of inflammatory signalling (52) MSC-sEVs can also specifically convert Th17 cells into ‘ex-Th17’ cells with low IL-17 secretion by degrading the RAR-related orphan receptor γt (RORγt) protein, thereby directly attenuating Th17-mediated inflammatory responses (53).

### 3.2 Inhibition of the proliferation and invasion of RA fibroblast-like synovial cells

Beyond their regulatory effects on circulating and localized immune cells, MSCs also directly target the pathogenic synovial stroma. The tumour-like abnormal proliferation and invasive migration of RA-FLS are key drivers of synovial vascularisation and progressive erosion of articular cartilage. MSCs and their derivatives can reverse the pathogenic phenotype of FLS through various mechanisms, including physical contact, environmental induction and nucleic acid delivery.

#### 3.2.1 The inhibitory effect of MSCs on RA-FLS

Once live MSCs have homing to the joint cavity, they can directly modulate the biological behaviour of RA-FLS by forming physical intercellular connections or releasing inhibitory paracrine signals (54). Studies have confirmed that, in animal models of RA treated with MSCs, reduced MMP expression and diminished cartilage erosion have been observed within the synovial tissue; this is closely associated with the inhibitory effect of MSCs on the pathological secretory function of RA-FLS (55).

#### 3.2.2 Pre-treatment strategies enhance the specificity of the inhibitory effect of MSCs

The inhibitory efficacy of MSCs against RA-FLS is significantly influenced by the dynamic regulation of the culture microenvironment. Experiments have shown that *in vitro* inflammatory pre-stimulation of MSCs with interferon-β (IFN-β) can significantly enhance their anti-invasive blocking capacity. MSCs activated by IFN-β pretreatment are able to more effectively inhibit the migratory activity of RA-FLS and substantially downregulate the expression of invasion and activation markers on their surface, providing a robust methodological approach for optimising MSC-based therapies through microenvironmental regulation in clinical settings (56). Furthermore, researchers have employed engineering approaches to enhance the functions of MSCs. For example, overexpression of CXCR7 improves the migratory and osteochondral differentiation capabilities of MSCs, whilst simultaneously enhancing their immunomodulatory activity against RA-FLS. Additionally, the combined use of MSCs with nanocarriers (such as liposomes and polymeric micelles) enables the targeted delivery of drugs or genes, thereby selectively inhibiting the pathogenic behaviour of RA-FLS (57).

#### 3.2.3 Molecular mechanisms underlying the targeted regulation of RA-FLS by MSC-EVs

EVs are efficiently taken up by aggressive RA-FLS and utilise the non-coding RNAs (circRNAs, miRNAs) they carry to top-down remodel the synovial disease network. On the one hand, via the miR-140-3p axis: Exosomes derived from human umbilical cord mesenchymal stem cells (HUCMSCs) are rich in miR-140-3p, a molecule that is efficiently taken up by RA-FLS. By targeting downstream signalling molecules, it inhibits the abnormal proliferation of FLS and the secretion of inflammatory factors, thereby alleviating the progression of RA (58). On the other hand, via the miR-100-5p/mTOR axis: small extracellular vesicles (sEVs) derived from macrophages carry miR-100-5p, which is capable of targeting the mammalian target of rapamycin (mTOR) signalling pathway, thereby remodelling the RA microenvironment and regulating the biological behaviour of RA-FLS (59). Furthermore, analysis of the plasma exosome miRNA profile has revealed 14 abnormally expressed miRNAs (such as miR-146a and miR-155) in the plasma exosomes of RA patients; these miRNAs participate in the synovial immune-stromal dialogue by regulating T-cell differentiation, FLS migration and osteoclast activation (60).

### 3.3 Reduces osteochondral damage and promotes repair and regeneration

The pathological features of end-stage RA are characterised by excessive osteoclast activation and a complete decline in osteoblast and chondrocyte function, ultimately leading to irreversible bone erosion and joint deformity. Thanks to their ability to regulate metabolic balance, secrete protease inhibitors and their direct differentiation potential, MSCs demonstrate unique therapeutic advantages in the protection and repair of hard tissues.

#### 3.3.1 The bone-regenerative effects of MSCs differentiating into bone or cartilage

Trophic support and paracrine-driven osteochondral protection While MSCs inherently possess osteogenic and chondrogenic differentiation capacity *in vitro* (61), their direct structural engraftment within the highly inflamed RA joint is severely limited. Instead, current evidence strongly suggests that MSCs protect damaged joints primarily by secreting a rich array of trophic factors and extracellular matrix (ECM) regulators. Rather than acting as direct building blocks, MSCs alter the local metabolic microenvironment to provide critical trophic support to endogenous chondrocytes and osteoblasts, thereby indirectly facilitating the intrinsic repair of articular cartilage defects (62) and mitigating the progressive bone erosion characteristic of RA. The differentiation and activity of osteoclasts (OCs) are primarily regulated by RANKL and its decoy receptor OPG. An increase in the RANKL/OPG ratio is a key event in bone resorption. Several studies have shown that MSC intervention can reduce the RANKL/OPG ratio, thereby inhibiting the survival, proliferation and differentiation of osteoclasts (37).

#### 3.3.2 Paracrine regulation of the RANKL/OPG balance inhibits osteoclast activation

EVs secreted by MSCs are rich in miRNAs, proteins and lipids, and serve as important carriers of their paracrine functions. In the treatment of RA, MSC-derived EVs have been shown to reduce inflammation and inhibit bone resorption. For example, by genetically engineering MSCs to produce exosomes that highly express PD-L1 (Exo-PD-L1), and given that activated synovial T cells are a major source of RANKL—a key factor promoting osteoclast differentiation and bone resorption—Exo-PD-L1 can directly reduce RANKL production by inhibiting T-cell activity, thereby blocking the pathological process of bone erosion at its source (42) It is worth noting that MSC-EVs can also influence bone metabolism by regulating the RANKL/OPG axis. For instance, in osteoporosis models, MSC-EVs can increase OPG levels and reduce RANKL expression, thereby protecting bone mass (63).

## 4 The tripartite dialogue: gut microbiota, MSCs, and the immune system – implications for RA therapy

MSCs and their EVs have demonstrated significant efficacy in regulating systemic immunity and protecting joints; however, the complete mechanism by which they initiate systemic immune tolerance and mediate signal transduction following their entry into the body has not yet been fully elucidated. In recent years, the gut-joint axis has attracted widespread attention as a key component in the pathogenesis of RA. This pathway reveals a bidirectional regulatory relationship between the gut microbiota, mucosal barrier function and peripheral joint inflammation (64). However, current research progress remains limited to correlational analysis; further studies are required to elucidate the underlying mechanisms and provide a focused synthesis of these findings. Here, we evaluate existing research and propose hypotheses regarding the underlying microscopic mechanisms.

### 4.1 The association between gut microbiota dysbiosis and the onset of RA

The composition of the gut microbiota in RA patients differs significantly from that in healthy individuals; this dysbiosis can increase intestinal mucosal permeability, allowing bacterial components such as lipopolysaccharides and metabolites to enter the circulation, thereby triggering a systemic immune response (65). A large number of clinical samples and multi-omics studies have revealed that the gut microbiota of RA patients is already significantly disrupted in the early stages of the disease, even before obvious symptoms appear. For example, compared with healthy individuals, the proportion of Prevotella copri in the intestines of newly diagnosed RA patients is as high as 75%, and its abundance is directly correlated with disease severity (66). Dysbiosis (such as P. copri overgrowth) can compromise intestinal epithelial integrity, increase intestinal permeability, allowing bacterial antigens or metabolites to enter the circulation and activate a systemic immune response. Furthermore, this bacterium promotes the differentiation of pro-inflammatory T helper 17 (Th17) cells and follicular helper T (Tfh) cells within the gut, thereby exacerbating systemic inflammation and autoantibody production (67). Furthermore, longitudinal studies have found that the gut microbiota profile in RA patients is not only associated with disease activity (e.g., DAS28 score) but may also predict treatment response (68). Consequently, the distinctiveness of the gut microbiota and its metabolites is undeniably significant, serving both as an emerging biomarker for the diagnosis of RA and as a key therapeutic target.

### 4.2 Interactions between MSCs and the gut microbiota in RA

Several studies have shown that the host microbiota (including the gut microbiota) can influence the self-renewal capacity, multipotent differentiation potential and immunomodulatory functions of MSCs (69). For example, the microbiota regulates the osteogenic differentiation of MSCs via metabolites or signalling pathways (70). Conversely, MSC transplantation itself can reshape the composition of the gut microbiota and the metabolite profile. In models of autoimmune diseases such as IBD and CD, MSC therapy significantly altered the diversity and abundance of the gut microbiota and modulated faecal metabolite pathways (67). Similarly, in a study using human amniotic mesenchymal stem cells (hAMSCs) to treat mice with type 1 diabetes, 16S rRNA sequencing revealed significant changes in the structure, diversity and abundance of the gut microbiota in the treatment group, accompanied by improved blood glucose levels and an increase in insulin-secreting cells (67). These findings suggest that MSCs may indirectly influence the progression of RA via the gut-joint axis: MSCs may modulate the gut microbiota—potentially restoring microbial homeostasis and reducing pathogenic bacteria, thereby indirectly mitigating joint inflammation in RA.

#### 4.2.1 Regulate the composition of the gut microbiota

Although direct microbiome-profiling data from MSC-treated RA patients remains limited, compelling evidence from other mucosal immune disorders demonstrates that MSC therapy can significantly ameliorate gut microbiota dysbiosis and reduce intestinal inflammation, e.g., in patients with IBD (71). Furthermore, MSC therapy may increase the abundance of bacteria that produce short-chain fatty acids (SCFAs). SCFAs (such as butyrate, propionate and acetate) are key metabolites produced by beneficial gut bacteria through the fermentation of dietary fibre; they play a role in maintaining intestinal barrier integrity, regulating immune tolerance and suppressing inflammatory responses (22). Drawing upon findings from other immune-mediated inflammatory models, we hypothesize that intravenous infusion of MSCs in RA patients holds the potential to influence the gut microbiota profile. If applicable to RA, this might attenuate the overgrowth of harmful bacteria such as *Prevotella copri*, whilst potentially increasing the abundance of beneficial bacteria capable of producing SCFAs. Consequently, this could limit the production of gut-derived inflammatory triggers at their source and potentially lower systemic inflammation driven by gut-derived antigens.

#### 4.2.2 Repair the intestinal mucosal barrier and prevent the translocation of endotoxins

MSCs possess inherent tissue repair and anti-inflammatory capabilities, enabling them to directly repair the damaged intestinal epithelial barrier. MSCs and their secretions release anti-inflammatory factors such as IL-10 and TGF-β, thereby improving the local immune status of the intestine; Preclinical evidence from models of intestinal injury suggests that MSC therapy is capable of mitigating damage to epithelial cells and promoting the restoration of tight junction structures, which could theoretically reduce intestinal permeability in the context of systemic autoimmune responses, thereby preventing inflammatory substances such as lipopolysaccharide (LPS) from entering the bloodstream. Furthermore, MSCs have been shown to repair chemically or radiation-induced damage to the intestinal barrier, alleviating pathological changes by upregulating tight junction proteins and anti-inflammatory pathways (72). We therefore hypothesise that, in the context of RA, MSCs can also exert their potent barrier-repairing effects and reduce intestinal permeability.

#### 4.2.3 MSC-EVs mediate gut-to-systemic immune regulation

MSC-EVs contain the PD-L1 protein and various immunoregulatory microRNAs, and are capable of precisely targeting gut-associated lymphoid tissue (GALT). We propose a theoretical model wherein MSC-EVs may initially interact with dendritic cells and T cells within the lamina propria of the intestinal mucosa, potentially facilitating the local generation of peripheral regulatory T cells (pTregs) in the intestine; It is therefore hypothesised that these anti-inflammatory immune cells, which are induced to mature in the gut, may enter the bloodstream via the mesenteric lymph nodes and potentially migrate to the synovial membrane of inflamed joints to exert anti-inflammatory effects. However, direct lineage-tracing evidence confirming the specific homing of gut-educated Tregs to RA joints is currently lacking, representing a critical gap that future spatial transcriptomics studies must address.

#### 4.2.4 The potential for combined treatment with gut microbiota interventions

Studies have found that natural substances such as Achyranthes bidentata polysaccharides can alleviate RA symptoms by modulating the gut microbiota, gut barrier and metabolites (73). This offers a treatment approach with practical application value: MSC therapy could be combined with probiotics, gut-modulating diets or targeted gut medications. By combining the immunomodulatory effects of MSCs with the gut-improving effects of microbiome interventions, a bidirectional synergistic effect could be achieved, thereby enhancing the efficacy of treatments targeting the gut-joint axis.

### 4.3 Limitations of current hypotheses and directions for future research

**Figure 2:**
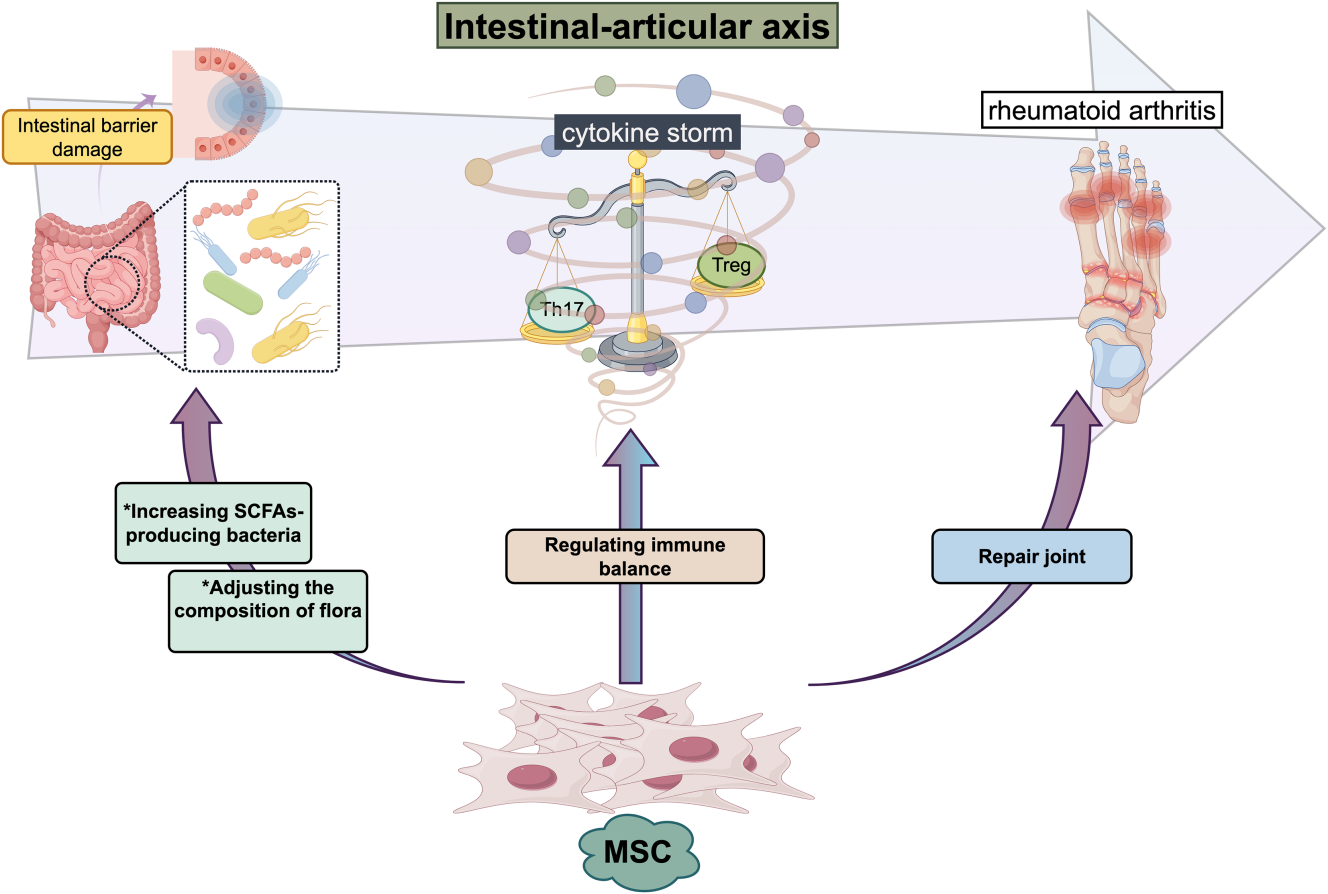
The proposed conceptual model of the gut-MSC-immune axis in RA. This schematic illustrates the hypothesized tripartite dialogue among gut microbiota, MSCs, and the immune system. It is important to note that while the individual components are supported by literature, the full causal chain depicted here represents an emerging theoretical model extrapolated primarily from inflammatory bowel disease (IBD) and general mucosal immunity research, which requires further *in vivo* validation specifically in RA models.

Although the aforementioned hypothesis regarding the treatment of RA by MSCs via the gut-joint axis is theoretically sound, there are currently significant shortcomings: there are no direct *in vivo* experiments or multicentre clinical data to demonstrate that MSCs treat RA by targeting the gut-joint axis. Most existing studies have focused solely on the systemic immunomodulatory functions of MSCs or have analysed the pathogenic role of the gut microbiota in RA; there has not yet been a systematic validation combining both aspects within a single arthritis animal model. Future research must delve deeper into the key molecular mechanisms underlying this relationship and identify the critical targets responsible for these differences. By combining the regenerative and immunomodulatory properties of MSCs, this approach could provide a powerful therapeutic tool for future precision medicine, potentially resolving current clinical bottlenecks in the treatment of refractory RA and addressing challenges such as the 50% failure rate observed with certain biologics.

(e.g., by increasing SCFA-producing bacteria) to support barrier restoration, and directly regulate immune balance by suppressing pathogenic Th17 responses (**Figure 2**).

## 5 Heterogeneity in clinical efficacy of MSC therapies

Although conventional disease-modifying antirheumatic drugs (DMARDs) and biologics remain the mainstays of clinical treatment for RA, they are often associated with a risk of infection and secondary failure. Over the past 15 years, MSC therapy has emerged as a highly promising alternative intervention, demonstrating generally good safety and varying degrees of clinical efficacy. **Table 1** provides a comprehensive summary of key clinical trials conducted in recent years. However, a critical review of these trials reveals significant heterogeneity in clinical outcomes (such as DAS28 and HAQ remission rates), necessitating a structured, in-depth interpretation based on cell source, dosing regimens and trial design.

**Table 1:** Overview of recent phase I/II clinical trials of mesenchymal stem cell therapy for rheumatoid arthritis.

Year/author	Phase	Patients (N)/design	MSC source	Dose & regimen	Follow-up	Key efficacy outcomes	Safety profile	Ref
2013 Wang et al.	I/II	172 (136 Tx, 36 Ctrl); RCT	UC-MSC	4.0×10^7^ cells, single IV; retreatment subgroups	12 months	↓DAS28/HAQ in retreatment group; improved pain/swelling at 2 weeks	Transient fever and chills	(74)
2017 Álvaro-Gracia et al.	I	16; open-label single-arm	UC-MSC	1×10^6^ cells/kg, single IV	6 months	↓DAS28, tender/swollen joint counts	Mild transient fever	(75)
2018 Park et al.	Ia	9; open-label single-arm	UCB-MSC	Dose-escalation, single IV	6 months	Dose-dependent ↓DAS28/VAS	No DLT; mild hyperuricemia	(76)
2018 Shadmanfar et al.	I/II	32 (16 Tx, 16 Ctrl); RCT	UC-MSC	2×10^7^ cells, single IV	12 months	Sustained ↓DAS28; reduced pro-inflammatory cytokines	No serious immune-related AEs	(77)
2019 Wang et al.	I/II	13; open-label single-arm	BM-MSC	1×10^6^ cells/kg, single IV	6 months	↓DAS28 (5.56→4.72); ↑FOXP3, IL-10, TGF-β1	No severe AEs	(78)
2020 Ghoryani et al.	I/II	105 (52 Tx, 53 Ctrl); RCT	UC-MSC	1.0×10^6^ cells/kg/wk ×4, IV	12 months	28/52 responders; peripheral immune remodeling	Well-tolerated	(79)
2020 He et al.	II	24; open-label single-arm	BM-MSC	1.5×10^6^ cells/kg, single IV	8 months	Improved ACR20/50 response rates	Generally safe	(80)
2020 Qi et al.	I/II	40 (20 Tx, 20 Ctrl); RCT	UC-MSC	1×10^6^ cells/kg, biweekly ×3	12 months	Higher ACR50/70 in MSC group	Mild infusion-related reactions	(81)

Firstly, the tissue origin of MSCs is a key biological factor driving clinical heterogeneity. As shown in **Table 1**, UC-MSCs are currently the most widely studied lineage in clinical research (e.g., Wang et al., He et al., Qi et al.). UC-MSCs have consistently demonstrated a strong capacity for inducing systemic immune remodelling. For example, a large-scale RCT (n=172) conducted by Wang et al. showed that UC-MSC infusion significantly reduced DAS28 scores and joint swelling, accompanied only by mild, transient fever. Similarly, umbilical cord blood-derived mesenchymal stem cells (UCB-MSCs) evaluated by Park et al. in a Phase Ia trial demonstrated a dose-dependent reduction in DAS28 and VAS pain scores. In contrast, bone marrow-derived mesenchymal stem cells (BM-MSCs) are typically preferred for highly refractory cases. Ghoryani et al. administered BM-MSCs ($1 \times 10^6$ cells/kg) to 13 women with refractory RA, significantly upregulating the expression of anti-inflammatory genes (FOXP3, IL-10, TGF-β1) and achieved sustained DAS28 remission at 6 months, suggesting that BM-MSCs possess unique mechanistic characteristics favouring long-term immune tolerance and local structural protection. Furthermore, adipose-derived MSCs (AD-MSCs) have shown well-tolerated safety in multicentre settings (Álvaro-Gracia et al.), and intra-articular delivery of BM-MSCs has emerged as an effective local intervention (Shadmanfar et al.).

Secondly, differences in dosing regimens have a significant impact on the duration of therapeutic efficacy. Early clinical trials typically relied on a single high-dose intravenous infusion. Although this approach is well tolerated, the immunomodulatory effect of a single dose may wane over time in the context of chronic, severe autoimmune disease. This was confirmed by Wang et al.: compared with patients receiving a single dose, those receiving repeated maintenance therapy at intervals (3, 6 or 8 months) exhibited a more stable and sustained reduction in disease activity. Consequently, recent trial designs have shifted markedly towards repeated, low-dose administration in divided doses. For example, Álvaro-Gracia et al. employed a fractionated regimen (infusions on days 1, 8, and 15) which was generally well tolerated. Furthermore, recent trials have explored combination strategies to enhance efficacy, such as integrating UC-MSCs with IFN-γ (He et al.), which yielded an ACR20 response rate of over 90% at 3 months. This highlights that sustained or pulsed MSC interventions may be essential for overcoming the relapsing nature of RA.

Finally, the reported efficacy must be interpreted objectively from the perspectives of trial design and patient baseline characteristics. The studies listed in **Table 1** span a wide range of evidence levels. Early open-label, single-arm studies (such as those by Park et al., Ghoryani et al.) were crucial for establishing safety and dose tolerability (Phase I/Ia); however, due to potential placebo effects and the lack of strict parallel controls, their efficacy data must be interpreted with caution. In contrast, Phase I/II randomised controlled trials (RCTs) (e.g., Wang et al., Álvaro-Gracia et al., Shadmanfar et al.) provide more rigorous, placebo-controlled evidence of efficacy. Looking ahead, accurately interpreting and enhancing the efficacy of MSCs urgently requires standardisation of these clinical variables, driving a steady transition in translational medicine from single-arm exploratory trials to multicentre RCTs stratified by specific pathological phenotypes.

## 6 Major challenges and future directions

**Figure 3:**
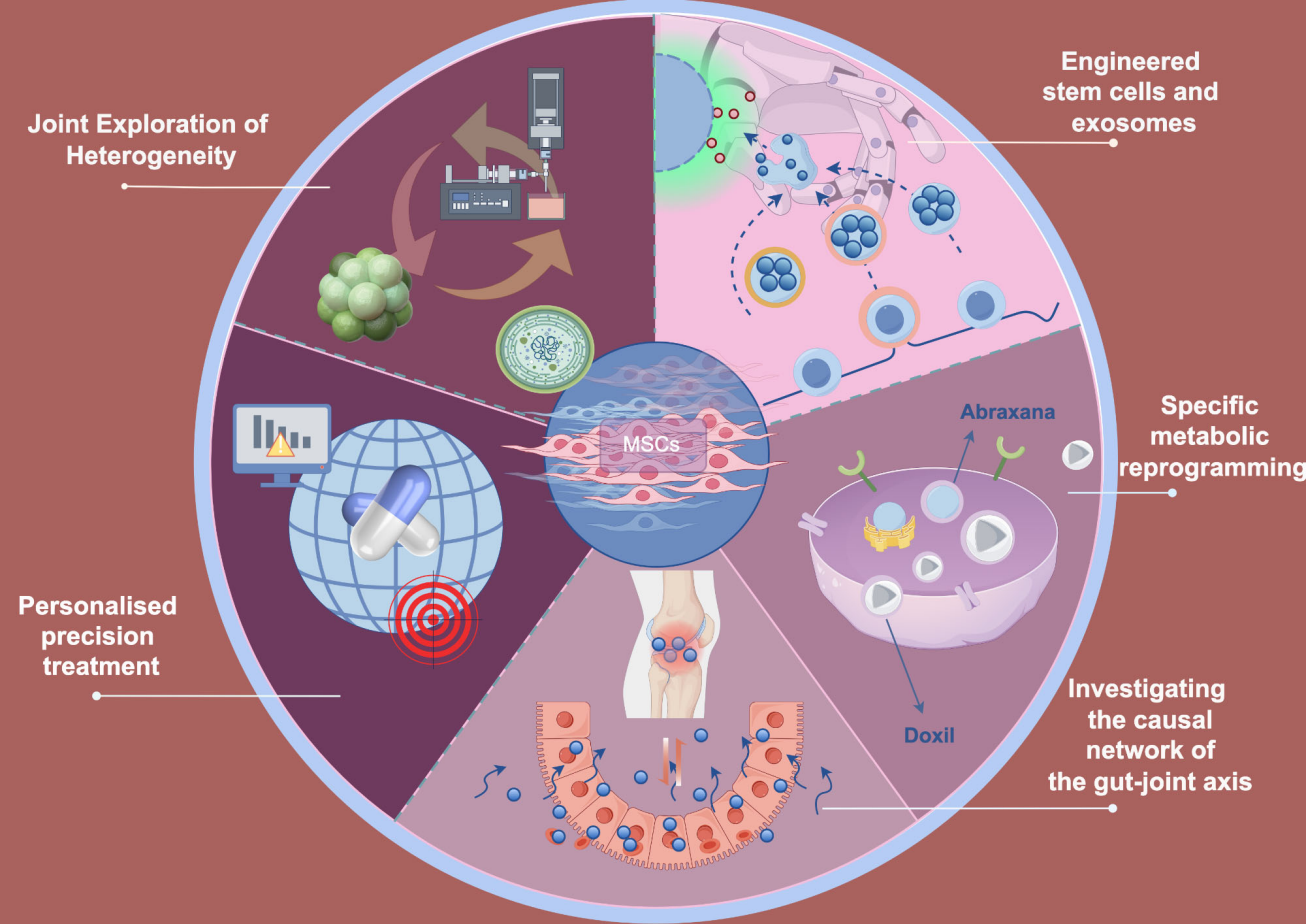
Future perspectives and key research directions for MSC-based therapies. This schematic outlines core strategies for advancing MSC research from mechanistic studies toward precision medicine. Key areas of focus include: (1) Causal network of the gut-joint axis: Shifting from correlational observation to causal validation of the ‘gut-MSC-immune’ network using germ-free models and multi-omics analyses; (2) Engineered stem cells and exosomes: Developing next-generation engineered MSCs and extracellular vesicles (EVs) via gene editing (e.g., CRISPR-Cas9) and cell-free nanodelivery platforms to precisely target specific pathological pathways; (3) Exploration of spatial heterogeneity: Employing single-cell and spatial multi-omics to map the *in situ* heterogeneity of the synovial microenvironment and MSC colonization at high resolution; (4) Specific metabolic reprogramming and personalized precision treatment: Integrating targeted drug delivery and systems biology to ultimately realize personalized therapeutic strategies for joint diseases.

The aforementioned microbiota-MSC combination therapy is a promising strategy to address the major challenges in the clinical translation of MSC-based therapy for RA, such as therapeutic efficacy heterogeneity and poor MSC survival in the hostile synovial microenvironment. Although MSCs and their derivatives have demonstrated clear efficacy in systemic immune modulation and local joint protection in RA, future translational research must move beyond mere ‘phenomenological observations’ and precisely target the specific pathological mechanisms and clinical translation bottlenecks identified in this review. Given the current gaps in the literature, future breakthroughs should focus on the following five areas (**Figure 3**).

### 6.1 Validating the causal network and microbiome synergy of the ‘gut-MSC-immune’ axis

With regard to the emerging conceptual model of the ‘gut-joint axis’ proposed in this review, the primary task for future research is to move from ‘correlational inference’ to ‘causal validation’. Studies should incorporate germ-free mouse models or faecal microbiota transplantation (FMT) techniques to directly verify whether the efficacy of MSCs depends on the presence of specific gut microbiota. Furthermore, by combining metagenomics with non-targeted metabolomics, the concentration dynamics of gut-derived metabolites (such as SCFAs or tryptophan metabolites) in synovial fluid following MSC administration should be tracked to elucidate precisely how MSCs block the migration of gut-derived antigens to the joint site by modulating the mucosal barrier.

### 6.2 Next-generation engineered MSCs and EVs targeting specific pathogenic mechanisms

As previously established, the late-stage destruction in RA is highly dependent on the dysregulation of the RANKL/OPG axis and the malignant proliferation of RA-FLS. Future cell engineering should not be limited to the use of native MSCs, but should instead focus on developing engineered formulations targeting specific pathological pathways. For example, utilising CRISPR-Cas9 technology to knock out specific kinases (such as CDK8) in MSCs that mediate the production of endogenous RANKL, thereby achieving a more potent osteoprotective effect; or utilising *in vitro* loading techniques to enrich MSC-EVs with specific non-coding RNAs (such as circFBXW7 or miR-34a) that possess anti-FLS proliferative and anti-apoptotic activities, thereby developing next-generation cell-free nanodelivery platforms that precisely target the ‘synovial-bone’ interface.

### 6.3 Analysing *in situ* spatial heterogeneity using single-cell and spatial multi-omics approaches

Given the high heterogeneity of RA-FLS across different anatomical sites and the uncertainty surrounding MSC colonisation sites, future mechanistic studies must evolve towards single-cell resolution. By combining single-cell RNA sequencing (scRNA-seq) with spatial transcriptomics, it is possible to precisely map the spatial distribution trajectories of MSCs or their vesicles within the proliferating synovium following their introduction into the body. This will help elucidate how MSCs engage in ‘single-cell-level’ *in situ* physical contact and paracrine interactions with specific synovial macrophage subpopulations (such as pro-inflammatory M1-like receptor subpopulations) or invasive FLS subpopulations, thereby revealing their precise immune remodelling networks within the microenvironment.

### 6.4 Metabolic priming in response to specific microenvironments

Given the harsh microenvironment within the RA joint cavity, characterised by extreme hypoxia, high lactate levels, nutrient deprivation and high oxidative stress (which are the key drivers of early apoptosis in transplanted cells), future cell pre-treatment strategies should move beyond the activation of a single inflammatory factor (such as IFN-β). Exploring specific metabolic reprogramming and fitness modification (such as short-term hypoxia pre-treatment, small-molecule activation of the glycolytic pathway, or specific regulation of mitochondrial autophagy) to enhance the *in situ* survival rate and paracrine efficacy of MSCs and their derived EVs when faced with severe metabolic stress represents a key biological approach to overcoming the current therapeutic bottleneck.

### 6.5 Focus on treatment heterogeneity: biomarker-based patient stratification and source matching

As demonstrated by the clinical evidence presented earlier (**Table 1**), there is significant heterogeneity in the efficacy of MSC therapy for RA; therefore, treating it as a universal ‘panacea’ is inconsistent with the principles of translational medicine. Future multicentre clinical trials must focus on identifying baseline serum or synovial fluid biomarkers that predict treatment response. By precisely stratifying RA patients (for example, accurately identifying ‘inflammation-dominant’ versus ‘bone erosion-dominant’ phenotypes), clinical decision-making can be guided as follows: For the former, umbilical cord-derived MSCs (UC-MSCs), which possess stronger systemic immunomodulatory capabilities, should be prioritised; whereas for the latter, bone marrow-derived MSCs (BM-MSCs), which excel in hard tissue protection and OPG secretion, should be precisely matched. This will ultimately lead to mechanism-driven precision cell therapy.

## 7 Conclusion

In conclusion, MSCs, by virtue of their dual capabilities in immunomodulation and tissue repair, offer transformative promise for RA therapy. This review outlines the multifaceted mechanisms of MSC action within the joint and highlights the profound, bidirectional ‘dialogue’ between MSCs and the gut microbiota. The proposed ‘gut-MSC-immune’ axis reframes RA from a disorder confined to the joints to a state of systemic immune-metabolic network dysregulation. This framework provides a novel theoretical basis for understanding interpatient therapeutic efficacy heterogeneity and identifies microbiota-MSC combination therapy as a highly promising future therapeutic direction. While challenges remain in standardization, safety assurance, and personalization of treatment regimens, interdisciplinary and rigorous clinical investigations enable MSC-based therapies—especially in synergy with microecological interventions—to pave a new path toward sustained remission and functional restoration in RA.
